# First person – Victor Tapia

**DOI:** 10.1242/dmm.052476

**Published:** 2025-05-23

## Abstract

First Person is a series of interviews with the first authors of a selection of papers published in Disease Models & Mechanisms, helping researchers promote themselves alongside their papers. Victor Tapia is first author on ‘ The role of 25-hydroxycholesterol in the pathophysiology of brain vessel dysfunction associated with infection and cholesterol dysregulation’, published in DMM. Victor is a postdoctoral research associate in the lab of Catherine Lawrence and Paul Kasher at The University of Manchester, Manchester, UK, investigating the causes and neuroprotective mechanisms of intracerebral haemorrhage using *in vitro* and animal experimental models.


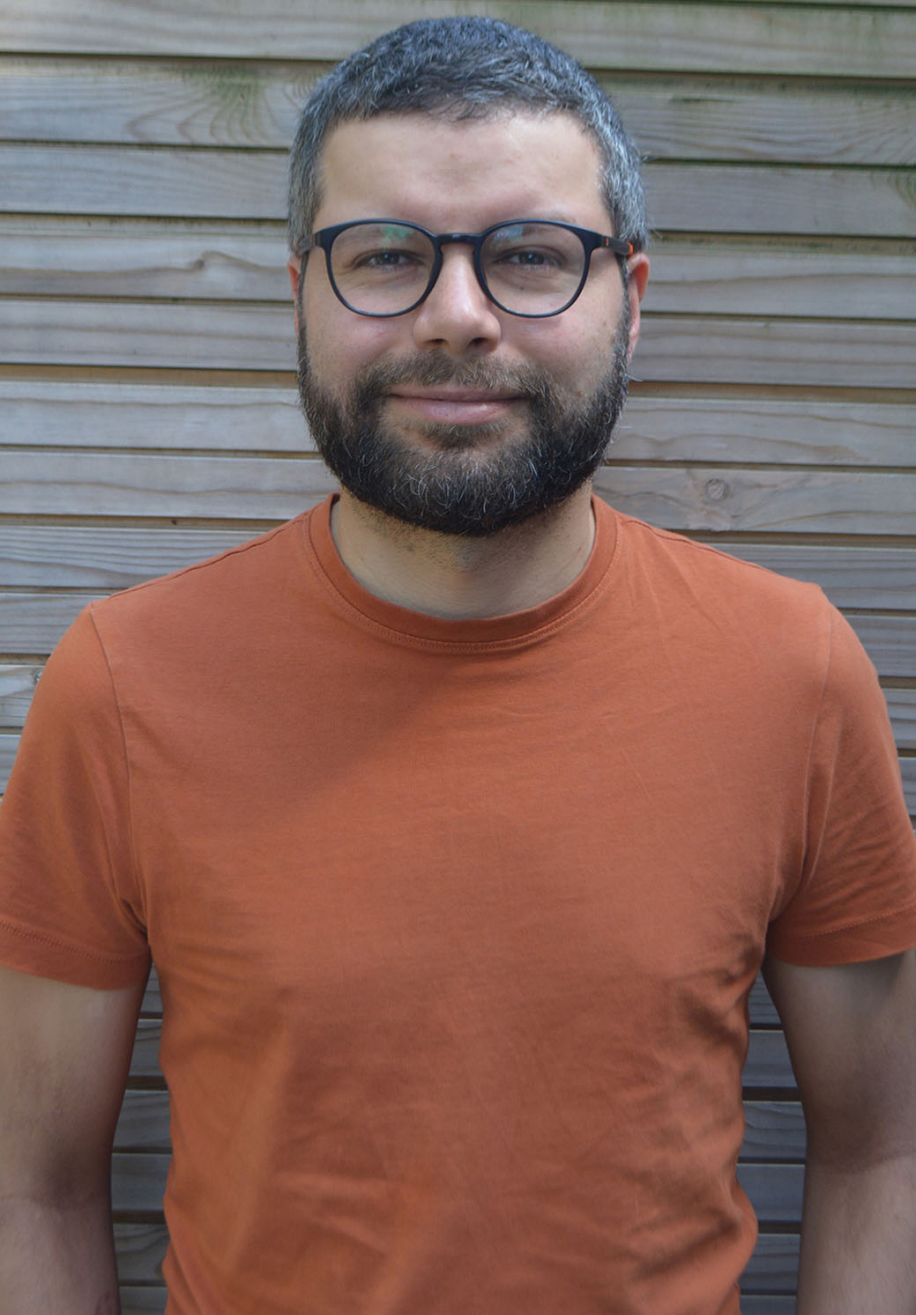


**Victor Tapia**

**Who or what inspired you to become a scientist?**

I've been drawn to the natural sciences since childhood, and my family – especially my father, who was a science teacher – really nurtured that curiosity. Studying biochemistry at university helped me narrow my focus to cellular biology, but it was during an undergraduate placement in a *Xenopus* research lab that everything clicked. That experience convinced me to pursue a scientific career and sparked my lasting interest in neuroscience and animal models.

Our study shows that brain endothelial cells are particularly sensitive to disruptions in cholesterol balance, suggesting that antiviral responses may unintentionally contribute to vascular damage through the CH25H/25HC pathway.

**What is the main question or challenge in disease biology you are addressing in this paper? How did you go about investigating your question or challenge?**

Intracerebral haemorrhage (ICH) is a severe type of stroke caused by the rupture of brain blood vessels. In our lab, we try to understand how different risk factors, viral infections in this study, contribute to the dysfunction and rupture of these vessels. Although viral infections have been proposed as a risk factor for ICH, the specific antiviral pathways involved remain unclear.

We focused on the enzyme cholesterol 25-hydroxylase (CH25H) and its metabolite, 25-hydroxycholesterol (25HC), which are part of an antiviral pathway that also regulates cholesterol metabolism. To investigate their role in ICH, we used zebrafish larvae, which are transparent, rapidly develop a complex nervous and vascular system, and offer well-established models of spontaneous brain haemorrhages.

Using a zebrafish model of coronavirus disease (COVID-19) cytokine storm, we found that CH25H expression increases in association with severe acute respiratory syndrome coronavirus 2 (SARS-CoV-2)-linked brain bleeding. We confirmed this observation in human foetal tissue with evidence of SARS-CoV-2-associated brain haemorrhages. Finally, we used a human brain endothelial cell line (hCMEC/D3) and zebrafish to test the effects of 25HC. We found that 25HC worsens brain bleeding in zebrafish and promotes endothelial dysfunction *in vitro*, through mechanisms dependent on cholesterol metabolism.

**How would you explain the main findings of your paper to non-scientific family and friends?**

We're trying to understand why blood vessels in the brain sometimes stop working and break, which can lead to a serious condition called haemorrhagic stroke. Some studies suggest that viral infections might make this worse, but we don't yet know how. In our study, we used zebrafish, a simple and useful model to study haemorrhagic stroke, and cells grown in the lab to look at how a specific antiviral response may lead to damage of brain blood vessels. We also confirmed some of our findings in human foetal brain tissue that showed signs of both viral infection (SARS-CoV-2) and brain bleeding. These findings help us start to piece together how infections might contribute to this kind of brain injury.

**What are the potential implications of these results for disease biology and the possible impact on patients?**

The CH25H/25HC pathway is well known for its role in antiviral defence through the regulation of cholesterol metabolism. However, its impact on vascular disease is only beginning to be understood. Our study shows that brain endothelial cells are particularly sensitive to disruptions in cholesterol balance, suggesting that antiviral responses may unintentionally contribute to vascular damage through the CH25H/25HC pathway. This has important implications, as low cholesterol levels (hypocholesterolaemia) and cholesterol-lowering treatments like statins have also been debated as potential risk factors for ICH. Our findings highlight a need to better understand how cholesterol metabolism affects brain blood vessels, which could ultimately guide safer treatment strategies and improve outcomes for patients at risk of haemorrhagic stroke.

**Figure:**
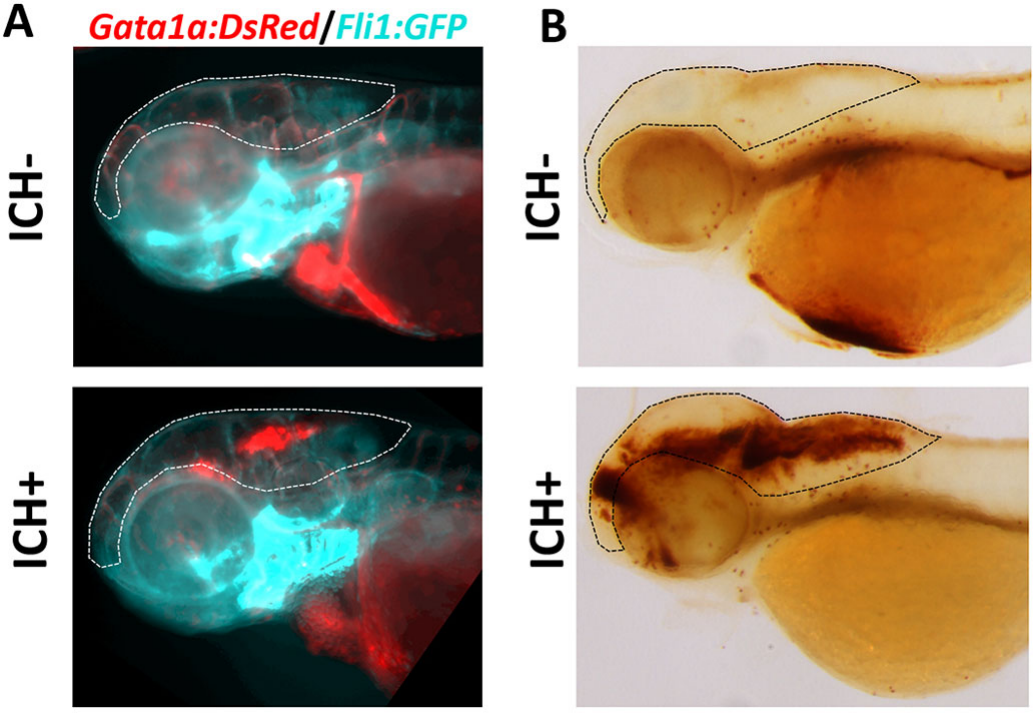
**Intracerebral haemorrhage (ICH) in zebrafish larvae.** (A) ICH observed using a red blood cell (*gata1a*^+^) fluorescent reporter. (B) ICH observed using o-Dianisidine staining. Dashed lines outline the brain area.

**Why did you choose DMM for your paper?**

We chose DMM because it's a high-quality, open-access journal with a clear focus on using model systems to understand disease mechanisms. Given that our study integrates multiple experimental models to investigate mechanisms of stroke, DMM felt like the ideal platform to share our findings with a broad and relevant audience.

**Given your current role, what challenges do you face and what changes could improve the professional lives of other scientists in this role?**

One of the main challenges as a postdoc has been balancing dedicated research time with the increasing demands of mentoring, management and faculty work. While I genuinely enjoy these aspects of the role, there's still a strong emphasis on scientific publication output as the primary measure of productivity. Another significant challenge is securing funding, particularly for early-career researchers who are non-citizens, as we often face additional visa and financial constraints. More flexible funding schemes and institutional support for balancing these roles would greatly improve the professional lives of researchers in similar positions.

**What's next for you?**

I'm currently working on a new research project that continues my focus on ICH, but this time using a mouse model to explore potential neuroprotective treatments.

**Tell us something interesting about yourself that wouldn't be on your CV**

Another scientific path I might have pursued is ecology, but I've kept that interest as a hobby. I'm really into hiking, birdwatching and nature photography. It's a great way to recharge and stay connected to the natural world outside the lab.

## References

[DMM052476C1] Tapia, V. S., Withers, S. E., Zhou, R., Bennington, A., Hoyle, C., Hedley, F., El Khouja, A., Luka, N., Massimo, M., Crilly, S. et al. (2025). The role of 25-hydroxycholesterol in the pathophysiology of brain vessel dysfunction associated with infection and cholesterol dysregulation. *Dis. Model. Mech.* 18, dmm052145. 10.1242/dmm.052145 40406995 PMC12128615

